# How Risk Signaling Influences Binge Drinking Impression Formation: An Evolutionary Experimental Approach

**DOI:** 10.3390/ijerph18115803

**Published:** 2021-05-28

**Authors:** Jordane Boudesseul, Oulmann Zerhouni, Laurent Bègue

**Affiliations:** 1 Instituto de Investigación Científica, Facultad de Psicología, Universidad de Lima, Av. Javier Prado Este 4600, Lima 33, Peru; 2 Laboratoire Parisien de Psychologie Sociale, University Paris Nanterre, 92000 Nanterre, France; oulmann.zerhouni@u-paris10.fr; 3 Laboratoire Inter-Universitaire de Psychologie, University Grenoble Alpes, 38400 Saint-Martin-d’Hères, France; laurent.begue-shankland@univ-grenoble-alpes.fr

**Keywords:** binge drinking, high school students, sexual selection theory, impression formation, sex differences

## Abstract

*Background*. Evolutionary theory-driven alcohol prevention programs for adolescents are lacking. This study introduced a binge drinking impression formation paradigm to test whether emphasizing sexual dysfunction induced by alcohol abuse lowers positive attitudes and expectancies related to binge drinking when compared with cognitive or long-term health consequences. *Method*. In a between-subjects experiment, 269 French high school students (age, *M* = 15.94, *SD* = 0.93, 63.20% women) watched professional-quality videos emphasizing sexual impotence (*n* = 60), cognitive impairment (*n* = 72), or long-term effects (cancer, cardiovascular disease, *n* = 68) induced by alcohol and then had to evaluate a drinking scene. We predicted that the video on impotence would be the most impactful when compared with the other videos. *Results*. Results showed that women evaluated the target as less attractive after viewing the cognitive video compared with the video on impotence. Men were more willing to play sports against the target after viewing the cognitive video, compared with the video on impotence. *Conclusions*. These results showed that evolutionary meaning might shape impressions formed by participants depending on the context. This study calls for further replications using the same design and materials.

## 1. Introduction

Alcohol is a harmful substance per se and has a direct impact on many risky related behaviors (sexual conduct, suicide, and road traffic injuries) and the World Health Organization considers alcohol production and consumption as highly relevant for the 2030 Agenda for Sustainable Development [1]. Individuals over 15 years old have seen their alcohol per capita consumption increased from 5.5 liters of pure alcohol in 2005 to 6.4 in 2016 with a recent decrease in Western Europe and the U.S. [2,3].

### 1.1. Social Theories of Binge Drinking

Past decades have seen the emergence of numerous social-psychological theories of binge drinking. Oei and Morawska [4], for instance, proposed the use of a cognitive framework based on alcohol expectancies and drinking refusal self-efficacy to explain four drinking styles: social drinkers, binge drinkers, regular heavy drinkers, and alcoholics. Attitudinal approaches based on their claims regarding Ajzen’s [5] theory of planned behavior (TPB) have been used to evaluate how attitudes, social norms, and perception of control contribute to drinking intentions, explaining up to 69% of the variance in some studies [6,7]. Other theories based on social bonding [8] or self-control [9] used inhibitory capacities, peer pressure, or environmental factors to explain binge drinking intensity and frequency. Although these are interesting theories to explain variations in binge drinking, they fail to account for binge drinking initiation and distal causes, have drawn numerous critiques (for critiques of TPB, see [10]), and have limited effectiveness in changing behavior (*d* = 0.14 to 0.68 with massive heterogeneity, see [11]).

### 1.2. Costly Signaling Theory

In the present study, we used an evolutionary framework to differentiate the risks at play in binge drinking videos. Costly signaling theory [12,13] proposes that specific messages are sent throughout the lifetime of an animal or individual and that context-specific responses may trigger a special type of behavioral response. In such a specific framework, binge drinking tends to fit in very well with Grafen’s criteria: the true genetic quality of the drinker, his advertising level, and his perceived value [14]. Some psychological evidence indicates that drinking quantity, frequency, and tolerance could be a signal of genetic quality [15,16,17]. For instance, Vincke [18] found that Flemish women evaluated both occasional and frequent drinkers as more attractive than non-drinkers. In another sample, Vincke [19] found that participants exposed to primes related to short-term motives (i.e., a casual relationship story) showed an increase in mating motivations, leading to the desire for higher alcohol amounts (without moderated effects). Highlighting the risk of sexual impotence should lead to a decrease in positive attitude when evaluating a binge drinker.

### 1.3. Short- vs. Long-Term Risks

The short- and long-term risks involved in binge drinking made our hypothesis compete with another one from a behavioral economic perspective. Tymula and colleagues [20] used an economic game to provide evidence for tolerance toward ambiguity in 33 adolescents (12–17 years old) compared with 32 adults (30–50 years old). They found that adolescents were more averse to clearly stated risks than older individuals. Adolescents were indeed more willing to tolerate ambiguous conditions when the probabilities of winning or losing were unknown. The short-term risks presented in our videos (e.g., sexual impotence or cognitive impairments) could be described as unambiguous risks since they can be directly connected to alcohol use and abuse whereas long-term risks come from repeated exposure.

### 1.4. Present Study

In the present study, we developed professional videos that were shown to participants during different classes at a French high school in Grenoble (France). We predicted that the video on impotence would be the most impactful when compared with the other videos. Moreover, we expected that men would be less willing to be a teammate of the male target presented in the video after viewing the video on impotence, and would prefer to play against him in a sports competition when compared with the other video (and similarly, for the short- vs. long-term comparisons). The present project aims at developing specific binge drinking videos differing only on the type of risks to precisely manipulate the signal sent. The objective is to test whether cues of sexual impotence deteriorate more binge drinking impression formations when compared to cues on cognitive deficits or long-term diseases.

## 2. Methods

### 2.1. Design and Data

This is a randomized single-blind order study design in 13 different classes (eight 10th grade and five 11th grade classes) from a high school in Grenoble, France. A total of 269 participants were recruited during class sessions in Spring 2017. Groups of 60, 72, and 68 participants were made by watching the videos on impotence, cognitive impairment, and long-term risks, respectively. Additionally, a control group of 69 students did not view any of the videos (see Table 1 for complete descriptive statistics). Giving the sensible nature of the sample (i.e., underage participants), data on individuals’ sexual orientation were not collected. All the 269 students recruited in the selected classes participated in the study on a total of 640 students in the school. These participants were included because high school is the time period where binge drinking initiations occurred. 

Because of study constraints, a sensitivity power analysis was used to determine the minimal detectable effect (MDE). Giving an α level at 5%, 90% of statistical power, 269 participants and 4 groups (the three video and the control group), the MDE for a one-way ANOVA was of medium size (Cohen’s *f* = 0.23).

### 2.2. Material 

During Spring 2016, we made three different videos (supplementary material available at https://osf.io/dhk7j/ accessed on 26 May 2021) with the help of French high school students and a professional video company (B Production), focusing on a young man binge drinking during a social gathering. The main structure of each video was the same, with the primary difference being the consequence of binge drinking experienced by the young man. In the video on cognitive impairment, the adolescent woke up the day after a party and experienced memory loss/lack of attention, and therefore could not properly study for his exam, which was followed by a short message on the cognitive risks involved in binge drinking. In the video on impotence, the young man was not able to have sex with his girlfriend, which was followed by a short message on the connection between sexual impotence and binge drinking. These two videos constituted short-term risks as they generally occur within minutes or hours after a binge drinking episode whereas the third condition is a problem that occurs later in life. Finally, the video on long-term risks depicted him years later, in a hospital bed, after having developed a terminal disease, followed by a short message on the effects of alcohol toxicity on the heart and other vital organs.

Giving the sensitive nature of our sample, key digital dependent variables were created and adapted for French underage participants and were specific to men and women. After watching one of the three videos, women were presented with a picture of a man drinking with a woman (Manon) by his side, and men were shown a highly intoxicated man (Nicolas) playing a drinking game. Both genders were asked to picture themselves in the situation (“You would not like to be in the same situation as Nicolas”). Women were asked to describe Nicolas’s features (“Manon seemed to finally have a crush on Nicolas,” “Nicolas is pretty attractive”, α = 0.29), whereas men evaluated Nicolas’s competitiveness used as two different DVs (“You could participate in a sporting event on Nicolas’s team,” “If necessary, you could play against Nicolas in a sporting event”, α = 0.51).

Participants were also asked standard sociodemographic questions (age, sex), drinking habits (frequency and intensity), and understanding of the experiments (see Table 1 for a complete description). Then, participants were asked about the above-mentioned items on situations embodiments, reproductive success and attractiveness (for women), and teammates and competition (for men).

## 3. Procedure and Analytic Plan

The present study had a between-subjects design, using videos focused on sexual impotence, cognitive impairment, or long-term effects (e.g., cancer, cardiovascular disease) potentially induced by excessive alcohol consumption. During a class session, participants were told that the university was running a program aimed at preventing alcohol abuse among youth. The study was simple blind as participants were not aware of which group was the experimental one, but researchers present were aware of the different hypotheses and conditions. To test our hypothesis that exposing students to sexual risks will have more impact on the impression formation of a binge drinker, we used contrast analysis to compare different conditions while controlling for the other one [21]. This includes the use of one-way ANOVA where we compared a contrast of interest (e.g., the sexual condition vs. cognitive while controlling for the variance of the two other conditions) while controlling for another competing contrast (e.g., the sexual conditions vs. all the other groups). Normality tests (e.g., Shapiro-Wilk), as well as a descriptive procedure (residual distribution and QQ-plot) were conducted to ensure application conditions were met. Since our DVs were skewed, we systematically re-ran the analysis using transformed log data, which tend to display normal distributions.

We also conducted a multilevel analysis with the student as level-1 and the class as level-2 analysis. Given the nature of our experimental design (exposure to messages was not randomized by students but by class), it was particularly appropriate to use a multilevel linear regression [22]. The model used the fixed effects stated earlier and controlled the random errors at the second level (i.e., random intercepts for the classroom), even though we also examined the dependent variable at the first level (students, see [23]).

## 4. Results

### 4.1. Descriptive Results

Participants were first asked standard sociodemographic questions. We observed that participants in the long-term video group were slightly younger (15.36 years old) compared to the other groups (16.10–16.18, *p* < 0.001). However, there were no significant differences on gender distribution (*p* = 0.17) or drinking intensity and frequency between the different experimental conditions (*p’s* > 0.10). Participants, on average, reported understanding very well the different spots (*M* = 6.26, *SD* = 1.33 on a 1–7 scale), and no differences were observed between the understanding of the different spots (*p* = 0.49).

### 4.2. Situation Embodiments

Results revealed that women were only marginally more willing to imagine themselves in the same situation as shown in the picture after viewing the video on impotence (*M* = 4.78, *SD* = 2.28) compared with the other videos (*M* = 5.23, *SD* = 2.17; *t*(168) = 1.82, *p* = 0.07, η_a_^2^ = 0.02; the effect did not reach significance when excluding women who never consumed alcohol, *p* = 0.16). When comparing the videos on clear, short-term risks with the video on long-term risks, women were less willing to be in Nicolas’s situation after viewing the clear risks videos (*M* = 5.14, *SD* = 2.14) than the long-term ambiguous-risks video (*M* = 4.60, *SD* = 2.52; *t*(168) = 2.07, *p* = 0.04, η_a_^2^ = 0.02; the effect was no longer significant when excluding women who never used alcohol, *p* = 0.32). In a multilevel model, the effect was no longer significant, *t*(164) = 1.03, *p* = 0.30 (see Table 2).

### 4.3. Reproductive Success and Attractiveness (Women Only)

Women who viewed the video on impotence evaluated the target as less attractive (*M* = 1.67, *SD* = 1.14, *n* = 46) than women who watched the other videos (*M* = 1.81, *SD* = 1.37), *t*(168) = 2.43, *p* = 0.016, η_a_^2^ = 0.02 (with two outliers; we checked in every analysis for potential statistical outliers following the studentized deleted residual technics, indicating that a level greater than 4 is considered an outlier, see [24]). However, when the video on impotence was compared to the video on cognitive impairment (*M* = 1.51, *SD* = 0.98, *n* = 41), as was the case in the second contrast analysis, Nicolas was judged as even less attractive, *t*(164) = 2.39, *p* = 0.02, η^2^ = 0.04.). The effect stills hold in multilevel analysis, *t*(168) = 2.14, *p* = 0.03 (but was no longer significant when excluding non-drinkers, *p* = 0.85). No differences were observed when women evaluated the reproductive success of the target (*p* = 0.78). On the other hand, neither the reproductive success nor the attractiveness measures were shown to be significant when comparing the short- and long-term risks videos (*p* = 0.43 and *p* = 0.09, respectively; see Table 2 for means and standard deviations across conditions).

### 4.4. Teammates and Competition (Men Only)

When asked if they would want Nicolas as a teammate, male participants did not rate Nicolas differently after viewing the video on impotence compared with the other videos (*p* = 0.37). However, they did indicate they would be more willing to play against Nicolas after viewing the video on impotence (*M* = 4.64, *SD* = 2.30) versus viewing videos on cognitive impairment (*M* = 5.17, *SD* = 2.51) or long-term diseases (*M* = 3.38, *SD* = 2.61), or being in the control group (*M* = 4.71, *SD* = 2.60; *t*(98) = 2.47, *p* = 0.015, η_a_^2^ = 0.04). However, our second comparison also came out significant, indicating participants would rather confront Nicolas in competition after viewing the video on cognitive impairment than the video on impotence, *t*(98) = 2.61, *p* = 0.01, η_a_^2^ = 0.04 (the effect ceased to be significant when excluding male participants who never drank alcohol, *p’s* > 0.20). Multilevel analysis indicates the effect hold when taking into account the level 2 unit “classrooms” (*t*(98) = 2.52, *p* = 0.01 and *t*(98) = 2.66, *p* = 0.009, respectively). Neither the teammate nor the competition measures were shown to be significant when comparing the short- and long-term risks videos (*p* = 0.50 and *p* = 0.06, respectively, see Table 2 below).

## 5. Discussion

In the present study, we introduced an impression formation paradigm grounded in evolutionary psychology in order to develop a preliminary education campaign targeting harms associated with drinking that could theoretically cause downstream reductions in drinking. While several social theories have investigated the correlational nature of such behavior [4,6,7,25,26], to our knowledge, no evolutionary theory has been used to build an impression formation paradigm targeting binge drinking impression formation. Based on the costly signaling theory [12,13], we postulated that men tolerating alcohol sent signals to both potential mates and other male competitors. By doing so, they hoped to display their genetic quality to others, publicly exposing their body to toxic substances and health consequences.

*Situation embodiments.* The only significant result was women’s lower willingness to be in Nicolas’s situation after viewing the short-term risks compared to the long-term one. Men, on the other hand, did not display any difference between videos. One possible explanation is that women may be more sensitive to the prevention of negative results whereas men are more influenced by positives outcomes coming from switching habits. Giving we only presented negative consequences of binge drinking, women were more concerned about the risks whereas men were not influenced by them.

*Reproductive success and attractiveness (women only).* Women who viewed the video on impotence evaluated the target as less attractive than women who watched the other videos. This demonstrates that alcohol-led sexual powerlessness affects women’s impression formation of men’s attractiveness. However, the effect was too small to reach significance on reproductive success measures. A possible explanation was also the virtual character of the experiment (i.e., videos), and more ecological design (i.e., in-bar setting) may display an effect on reproductive success evaluation because of cues-dependent context (i.e., facial expressions, vocal signals, etc.).

*Teammates and competition (men only).* Finally, when asked if they would want Nicolas as a teammate, male participants did not respond differently after viewing the video on impotence, compared with the other videos. However, they did report they would rather confront Nicolas in the competition after watching the video on cognitive impairment than the video on sexual impotence. This may indicate that in a school context, cognitive cues are more reliable and better signal one’s genetic quality than sexual capabilities. A field study comparing these measures would be a good way to explore how much binge drinking impression formation is context-dependent.

## 6. Limitations 

As with any innovative program, our study had several limitations. First, we did not directly evaluate the participants’ evaluation of the main actor in the video for ethical and methodological reasons. We used an indirect impression formation task after participants watched a video, with our primary interest being to observe how risk signaling could impact the way individuals judge an intoxicated peer during a social gathering. Showing that specific signals impact impressions more than others could be of great help to the health and education community and contribute to a deeper understanding of binge drinking as a multi-faceted phenomenon. Further research should include impression tasks where the actors tolerate alcohol to further test Zahavian’s prediction.

One possible limitation to our videos was that a man who was not tolerant to alcohol sent de facto signals of low genetic quality to participants. To that extent, possible floor-effects could have emerged, making the target (Nicolas) unfit, whatever the situation following the video may be. Receiving short- or long-term signals, sexual or not sexual, would in that case make no difference regarding the target’s capabilities. Conversely, measuring the transfer of those signals to a man with a high alcohol sensitivity may show higher variations between videos, leading to more subtle measurements.

Another constraint is that our study did not directly test the impact of the different videos on future drinking intentions. It would be particularly useful to realize longer videos and evaluate whether exposure to such messages could help to decrease binge drinking frequency and intensity compared to other effective brief interventions such as theTertiary Health Research Intervention Via Email (THRIVE), the AlcoholEDU programs [27], and message framing [28,29]. Our sample was also mainly composed of underage participants, which makes it difficult to generalize to older populations such as college students. It would be particularly useful to see whether the different signals produce different impression formation among older drinking peers.

In addition, the number of participants who never drank alcohol was relatively high compared to the general population, and some findings were no longer significant when excluding them. This calls for a better understanding of the difference between drinkers’ and nondrinkers’ impression formation. Meta-analysis on brief interventions indicates modest but positive effects on the reduction of both alcohol and other drug use (Tanner-Smith et al., 2015). Further studies should explore the cognitive mechanism in the impression formation of binge drinking and other drugs to optimize the use of brief alcohol intervention.

## 7. Conclusions

The main objective of this study was to create evolutionary-based binge drinking videos to test whether different signals linked to alcohol excessive consumption might influence impression formation of a binge drinker. While some data confirmed our expectations, others did not. Women did evaluate as less attractive the binge drinker victim of sexual impotence compared to other groups, but this did not influence their evaluation of his reproduction success. On the other hand, male participants tend to be more confrontational against the actor with cognitive deficit than the sexually powerless one. These data suggest that binge drinking impression formation may also be context-dependent and that health information presented in a classroom does not probably encompass all social cues that are present during a drinking social event [30]. Collecting data in a field study to evaluate how real-time binge drinking cues impact impression formation could help generate a more effective evidence-based prevention program.

## Figures and Tables

**Table 1:** Demographics characteristics, drinking frequency and intensity and spot understanding comparisons between the sexual, cognitive, long-term and control conditions.

Variable	Sexual Condition (*n* = 60)	Cognitive Condition (*n* = 72)	Long-Term Condition (*n* = 68)	Control Condition (*n* = 69)	*p*-Value
Age, M (SD)	16.12 (0.88)	16.18 (0.85)	15.36 (0.61)	16.10 (1.07)	*p* < 0.001
Gender - Male - Female	14 46	31 41	26 42	28 41	*p = 0.17*
How many days in total have you drink alcohol? *(n, %)* *In your life* - *Never* - *1–2 days* - *3–5 days* - *6–9 days* - *10–19 days* - *20–29 days* - *30+ days* *In the past 30 days* - *Never* - *1–2 days* - *3–5 days* - *6–9 days* - *10–19 days* - *20–29 days* - *30+ days*	27 (47.40) 4 (7.00) 7 (12.30) 6 (10.50) 2 (3.50) 3 (5.30) 8 (14.00) 39 (70.90) 9 (16.40) 4 (7.30) 2 (3.60) 0 (0.00) 0 (0.00) 1 (1.80)	32 (47.80) 2 (3.00) 2 (3.00) 6 (9.00) 5 (7.50) 6 (9.00) 14 (20.90) 39 (59.10) 14 (21.20) 5 (7.60) 7 (10.60) 1 (1.50) 0 (0.00) 0 (0.00)	39 (58.20) 10 (14.90) 6 (9.00) 2 (3.00) 5 (7.50) 1 (1.50) 4 (6.00) 51 (81.00) 8 (12.70) 3 (4.80) 0 (0.00) 1 (1.60) 0 (0.00) 0 (0.00)	32 (49.20) 17 (26.20) 3 (4.60) 3 (4.60) 1 (1.50) 3 (4.60) 6 (9.20) 51 (81.00) 5 (7.90) 6 (9.50) 0 (0.00) 0 (0.00) 0 (0.00) 0 (0.00)	

					*p* = 0.13







					*p* = 0.41






*How many drinks do you usually have in a single event? (n, %)* - *None* - *<1 drink* - *1 drink* - *2 drinks* - *3 drinks* - *4 drinks* - *5+ drinks*	26 (43.30) 4 (6.70) 6 (10.00) 6 (10.00) 2 (3.30) 2 (3.30) 5 (8.30)	29 (40.28) 6 (8.33) 6 (8.33) 2 (2.78) 8 (11.11) 2 (2.78) 9 (12.50)	36 (52.94) 9 (13.24) 8 (11.76) 3 (4.41) 3 (4.41) 2 (2.94) 3 (4.41)	35 (50.72) 9 (13.04) 9 (13.04) 4 (5.80) 4 (5.80) 4 (5.80) 1 (1.45)	*p* = 0.16
*Spot understanding (M, SD)*	6.05 (1.43)	6.30 (1.49)	6.38 (1.16)		*p* = 0.49

Note. Mean comparisons based on one-way ANOVAs.

**Table 2:** Means and standards deviations of the different dependent variables across the different conditions.

Measures/Conditions (*M* (*SD*))	Sexual	Cognitive	Long-Term	Control	*ƞ_a_*^2^ (*p*-Value) *
Willingness to be in the target’s situation (men)	5.93 (2.13)	5.00 (2.51)	5.19 (2.55)	5.82 (2.25)	0.00 (0.49)
Willingness to be in the target’s situation (women)	4.78 (2.28)	5.54 (1.92)	4.60 (2.51)	5.59 (1.91)	0.02 (0.07)
You could be the target’s teammate (men)	2.71 (2.02)	2.76 (2.08)	2.27 (2.05)	2.67 (2.30)	0.00 (0.37)
You could play against the target (men)	4.64 (2.31)	5.17 (2.51)	3.38 (2.61)	4.71 (2.61)	0.04 (0.015)
Target’s reproductive success (women)	3.78 (1.79)	4.17 (1.75)	4.00 (1.93)	4.33 (2.07)	0.00 (0.78)
Target’s attractiveness (women)	1.67 (1.14)	1.51 (0.98)	2.19 (1.61)	1.71 (1.36)	0.02 (0.016)

Note. * Contrast analysis comparing the video on sexual impotence to the cognitive one while controlling for the other two conditions (long-term disease and control groups; see Brauer & McClelland, 2005).

## Data Availability

Data and material are publicly available at https://osf.io/dhk7j/, accessed on 26 May 2021.
